# Role of *de novo* cholesterol synthesis enzymes in cancer

**DOI:** 10.7150/jca.38598

**Published:** 2020-01-17

**Authors:** Jie Yang, Lihua Wang, Renbing Jia

**Affiliations:** 1 Department of Ophthalmology, Ninth People's Hospital of Shanghai, Shanghai Jiao Tong University School of Medicine, Shanghai, China.; 2 Shanghai Key Laboratory of Orbital Diseases and Ocular Oncology, Shanghai, China.

**Keywords:** metabolic reprogramming, *de novo* cholesterol synthesis, cancer progress

## Abstract

Despite extensive research in the cancer field, cancer remains one of the most prevalent diseases. There is an urgent need to identify specific targets that are safe and effective for the treatment of cancer. In recent years, cancer metabolism has come into the spotlight in cancer research. Lipid metabolism, especially cholesterol metabolism, plays a critical role in membrane synthesis as well as lipid signaling in cancer. This review focuses on the contribution of the *de novo* cholesterol synthesis pathway to tumorigenesis, cancer progression and metastasis. In conclusion, cholesterol metabolism could be an effective target for novel anticancer treatment.

## Introduction

Over the past few decades, numerous published studies have focused on cancer cell metabolism and have determined that metabolic reprogramming is a hallmark of cancer^1^-^6^. It is thought that reprogramming of catabolic and anabolic metabolism to harvest energy and synthesize biomass is critical for the survival and growth of cancer cells. Nearly a century ago, Otto Warburg observed that cancer cells tend to use glucose extensively through aerobic glycolysis^7^. Highly proliferative cancer cells also tend to have a high lipid content (fatty acids and cholesterol), which is important for providing energy, membrane synthesis, and lipid signaling^8^. Cancer cells often exhibit an enhanced ability to synthesize lipids and have a higher lipid uptake^9^. Most studies have reported that the upregulation of fatty acid and cholesterol related enzymes is required for tumor progression^9^-^14^. Lipid metabolism involves lipid synthesis, storage and degradation. In mammals, cholesterol is either absorbed from dietary sources or synthesized *de novo*. The liver and intestinal mucosa are the main sites of cholesterol synthesis. Up to 70-80% of cholesterol in humans is synthesized *de novo* by the liver, and 10% is synthesized *de novo* by the small intestine. Accumulating evidence demonstrates that cholesterol plays a critical role in cancer progression^15^-^19^. Furthermore, intracellular cholesterol homeostasis is different among various cancer types, and cholesterol itself plays varying roles among different cancer types 17. In this review, we describe normal cholesterol synthesis and cholesterol metabolic changes in cancer cells. Cholesterol biosynthesis pathways could be an attractive therapeutic target for cancer therapeutics.

## Total cholesterol and cancer

Cholesterol is a primary lipid that is essential for membrane biogenesis, cell proliferation, and differentiation. Cholesterol is also the precursor of steroid hormones and sterols that induce specific biological responses. Cholesterol is mainly synthesized by the liver in humans, and is distributed throughout the body via high-density lipoprotein (HDL) and low-density lipoprotein (LDL) transporters. Acetyl-CoA is a key precursor of *de novo* cholesterol synthesis 20. The reduction of HMG-CoA is an important regulatory step in cholesterol synthesis. Cholesterol itself is an important metabolic intermediate that is converted into cholesteryl esters, bile acids, cholecalciferol/vitamin D, and various steroid hormones in the appropriate tissues. Cholesterol biosynthesis, regulation of cholesterol plasma levels, and conversion to other compounds is normally carefully regulated 21. Unlike normal cells, tumor cells upregulate intracellular cholesterol synthesis and exhibit abnormal aggregation of most metabolites.

## Transcription factor and cholesterol *de novo* synthesis enzymes

**Figure 1:**
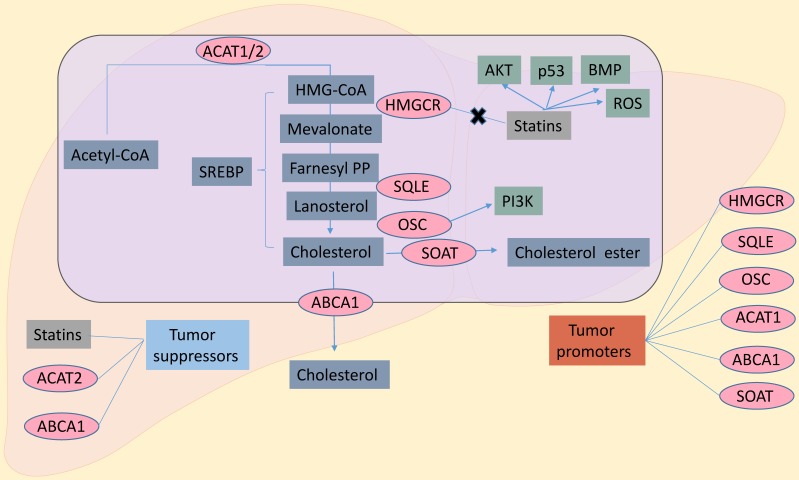
** Cholesterol biosynthesis pathway in cancer cells.** Inhibitors of HMGCR, statins could exert anti-cancer effects through AKT, p53, BMP, ROS. And OSC through PI3K promoted cancer growth. To sum up, HMGCR, SQLE, OSC, ACAT1, SOAT and ABCA1 are the contributing factors in cancers. Statins, ACAT2 and ABCA1 are inhibitors in cancers. SREBP, sterol regulatory element binding protein; ACAT1/2, acetyl-CoA acetyltransferase 1/2; SOAT, sterol-o-acyltransferase; HMGCR, hydroxy-3-methylglutaryl-coenzyme a reductase; SQLE, squalene epoxidase; OSC, oxidosqualene cyclase; ABCA1, ATP-binding cassette transporter A-1; PI3K, phosphatidylinositol 3-kinase; AKT, protein kinase B; ROS, reactive oxygen species; BMP, bone morphogenetic protein.

Several steps are required to convert acetyl-CoA to cholesterol, which then is involved in numerous biological roles. These steps involve cholesterol synthase (ACAT, HMGCR, SQLE, OSC), acyl coenzyme A, cholesterol acyltransferases (SOAT), and ATP-binding cassette transporter A-1 (ABCA1). In a situation of decreasing cholesterol availability, inhibiting these enzymes could influence cancer cell growth. Interestingly, many inhibitors of these enzymes have effects on cancer treatment (Figure 1). SREBPs, which were reported the most transcription factors (sterol regulatory element binding proteins,) regulate cholesterol *de novo* synthesis. Also, KLF14^22^, ChREBP^23^,^24^, LXRα^25^ and LRH-1^26^ have very important roles in cholesterol metabolism. Due to the limitation of words, we just reviewed the role SREBP played on it.

### SREBP

Lipid homeostasis in vertebrate cells is regulated by a series of membrane-bound transcription factors, the sterol regulatory element-binding proteins (SREBPs). SREBPs directly activate more than 30 genes specific to the synthesis and uptake of cholesterol, fatty acids, triglycerides, and phospholipids, as well as the nicotinamide adenine dinucleotide phosphate cofactor required to synthesize these molecules 27.

In 2016, Zhao et al. demonstrated that the hepatitis B X-interacting protein (HBXIP) upregulates SREBP-1c/SREBF1, which activates the transcription of fatty acid synthase by directly interacting with nuclear receptor coactivators and LXR. Overexpression of SREBP-1c can also activate HBXIP transcription. HBXIP enhances fat production, leading to the growth of breast cancer cells *in vitro* and *in vivo* 28. Therefore, direct inhibition of SREBPs, resulting in cholesterol depletion, may be an effective cancer therapeutic strategy 29.

### HMGCR

The 3-hydroxy-3-methylglutaryl-CoA reductase (HMGCR) enzyme is the rate-limiting enzyme of the cholesterol synthesis pathway. In 2016, two reports showed that autoantibody-positive anti-HMGCR myopathies and HMGCR-positive inflammatory myopathies were association with cancer 30,31. In a clinical study, positive cytoplasmic HMGCR expression was associated with colon cancer with distant metastasis-free disease at diagnosis. Positive HMGCR expression was significantly associated with prolonged cancer-specific survival in an unadjusted Cox regression analysis in the entire cohort and in stage III-IV disease 32. Increased HMGCR expression was also observed in gastric cancer tissues. Overexpression of HMGCR promoted the growth and migration of gastric cancer cells, while HMGCR knockdown inhibited growth, migration and tumorigenesis 33. Upregulation of HMGCR was also observed in clinical glioblastoma samples. Forced expression of HMGCR promoted the growth and migration of U251 and U373 cells, while knockdown of HMGCR inhibited their growth, migration and metastasis 34. HMGCR was overexpressed in prostate cancer (PC) stroma, especially in early-stage PC. These results provide insights into the molecular mechanisms underlying PC invasion 35. Another study found that ectopic expression of hsa-miR-195 in MCF-7 and MDA-MB-231 cells, resulting from targeting HMGCR, significantly altered cellular cholesterol and triglyceride levels as well as reduced proliferation, invasion and migration 36.

Statins are often used to lower cholesterol by inhibiting HMGCR. The multipotency of statins has been associated with cancer risk. Several studies have also found that statins play an important role in the treatment of several cancers 37. In 2012, Gazzerro et al. reviewed statin pharmacology, elucidating the prospect of utilizing statins in cancer treatment for hepatocellular carcinoma (HCC), colorectal carcinoma (CRC), and acute myelocytic leukemia 38. A recent Danish study correlated the use of statins in cancer patients. They observed reduced cancer-related mortality for 13 cancer types among statin users. Statin use in patients with cancer is also associated with reduced cancer-related mortality 39. In epithelial ovarian cancer, a retrospective study reported that the use of statins was associated with improved clinical outcomes 40. Statin use prior to a cancer diagnosis was correlated to a reduction in all-cause and cancer-specific mortality. Consistent with these data, patients with CRC with pre-diagnosis statin use had prolonged cancer-specific survival, but no benefits were observed for patients with post-diagnosis statin use 41. Additionally, the risk of CRC was lower in statin users versus nonusers 42. The anticancer effect of simvastatin through the induction of apoptosis is related to AKT signaling-dependent downregulation of survival in A549 lung cancer cells 43. In an analysis of 999 colon cancer patients, statin use was correlated with a reduced risk of death from any cause or from cancer. The benefit of statin use was greater for patients whose tumors had intact bone morphogenetic protein signaling independent of *KRAS* mutation status 44. Simvastatin also affected OCM-1 cell growth, apoptosis and cell cycle. In addition, simvastatin resulted in increased ROS levels and significantly increased apoptosis and the expression of the mitochondrion-related apoptosis protein p53 in OCM-1 cells 45. In 2016, a surprising report found that statins preferentially inhibited the growth of cancer cells that express mutations, and p53 status impacted statin-dependent efficacy of cancer therapy 46.

### ACAT

Acetyl-CoA acetyltransferase 1 (ACAT1) is a tetrameric enzyme in the ketogenesis pathway that converts two acetyl-CoA molecules into acetyl-CoA and CoA 47-49. Fan et al. found that knockdown of ACAT1 attenuated tumor growth 50. The inhibition of tetrameric ACAT1 by abolishing Y407 phosphorylation, or eliminating arecoline hydrobromide treatment, resulted in suppressed ACAT1 activity 51. This lead to increased pyruvate dehydrogenase complex flux and oxidative phosphorylation, with attenuated cancer cell proliferation and tumor growth. These findings suggested that ACAT1 could be an effective anticancer target. Acetyl-CoA acetyltransferase 2 (ACAT2) also plays an important role in lipid metabolism. ACAT2 downregulation has been associated with a poorer cancer-specific survival prognosis in clear cell renal cell carcinoma 52.

### SOAT

Sterol-o-acyltransferase (SOAT), also called Acyl-CoA cholesterol acyltransferase (ACAT), is an integral membrane protein of the rough ER that catalyzes the formation of cholesteryl esters from cholesterol and long-chain fatty acids. The solubility characteristics of cholesteryl esters make them the ideal molecule for cholesterol storage, which occurs mostly in cytoplasmic lipid droplets inside cells. Thus, SOAT is a key enzyme involved in the control of intracellular cholesterol storage and in determining free cholesterol levels 53. SOAT plays an important role in the homeostasis of cell cholesterol metabolism and is a drug target for treatment intervention in various diseases, such as atherosclerosis, Alzheimer's disease and cancer 54.

In 2019, through proteomic and phosphor-proteomic profiling, he's group found that SOAT1 expression was higher in HCC and was associated with a poor prognosis. In addition, inhibiting SOAT1 markedly suppressed cell proliferation and migration 55. SOAT1 was also overexpressed in human castration-resistant metastatic PC tissues. These results indicate that the enzymes involved in the ketogenic pathway are upregulated in high-grade PC and could act as potential tissue biomarkers for the diagnosis or prognosis of high-grade disease 56. Li et al. found an abnormal accumulation of cholesteryl ester in human pancreatic cancer specimens and cell lines, which was mediated by SOAT1. SOAT1 expression was associated with poor outcomes. By using an SOAT1 inhibitor or shRNA knockdown, abrogated cholesterol esterification significantly suppressed tumor growth and metastasis in an orthotopic pancreatic cancer mouse model. SOAT1 inhibition was found to increase intracellular free cholesterol levels, which was associated with ER stress and apoptosis 57. Furthermore, the availability of cholesteryl esters increased the proliferation and invasiveness of normal cells, indicating that cholesteryl esters may contribute to a tumor-promoting phenotype. Another study showed that breast cancer cell lines treated with auraptene, a naturally occurring SOAT1 inhibitor, also had decreased cellular proliferation, invasion, and colony formation 58. Treatment of human prostate, pancreatic, lung, and colon cancer cell lines with avasimin, a potent SOAT1 inhibitor, significantly reduced cholesteryl ester storage in lipid droplets and increased intracellular free cholesterol levels. This led to apoptosis and the suppression of proliferation. Systemic treatment of avasimin notably suppressed tumor growth in mice and prolonged survival time 59. Similarly, SOAT1 inhibition decreased cell proliferation and invasion in two tumor cell lines 60. The SOAT1 inhibitor CP-113818 reduced proliferation of breast cancer cells and specifically inhibited LDL-induced proliferation of ERα- cells. A greater ability to take up, store and utilize exogenous cholesterol conferred a proliferative advantage in basal-like ERα- breast cancer cells as well 61.

Revitalizing the cytotoxic potential of CD8+ T cells is of great clinical interest in the cancer immunotherapy field. A novel mechanism to enhance the antitumor response of CD8+ T cells in mice by regulating cholesterol metabolism has been reported. The inhibition of cholesterol esterification in T cells, either by genetic ablation or by pharmacological inhibition of SOAT1, resulted in enhanced effects and proliferation of CD8+ T cells but not CD4+ T cells. In the absence of SOAT1, CD8+ T cells performed better than wild-type CD8+ T cells in controlling the growth and metastasis of melanoma in mice. In addition, combination therapy with avasimibe, SOAT inhibitors and anti-PD-1 antibodies had a superior effect on controlling tumor progression than single agent therapy 62.

Human SOAT2 is mainly expressed in the intestine and fetal liver. Inhibiting SOAT2 leads to the intracellular accumulation of unesterified oxysterols and suppresses the growth of both HCC cell lines *in vitro* and as xenograft tumors. Further mechanistic studies have revealed that HCC-linked promoter hypomethylation is a major mechanism for *SOAT2* gene expression induction. Specifically blocking the cholesterol metabolism pathway established in HCC may have therapeutic effects in HCC patients 63. The inhibition of SOAT2 expression significantly decreased leptin-induced proliferation, migration and invasion of MCF-7 and T47D cells. Additionally, leptin may enhance the proliferation, migration and invasion of breast cancer cells in an SOAT2-dependent manner through the PI3K/AKT/SREBP2 signaling pathway 64.

### SQLE

Squalene epoxidase (SQLE) is involved in the first oxygenation step in the cholesterol synthesis pathway, and is therefore a good target for controlling the cholesterol synthesis process 65. Liu et al. sequenced RNA from nonalcoholic fatty liver disease (NAFLD)-induced HCC samples and revealed *SQLE* as the top metabolic gene overexpressed in NAFLD-induced HCC patients. In human NAFLD-induced HCC and HCC, *SQLE* was overexpressed and its expression was associated with poor patient outcomes. Terbinafine, an SQLE-targeting drug, markedly inhibited SQLE-induced cell growth in NAFLD-induced HCC and HCC cells and attenuated tumor development in xenograft models and SQLE transgenic mice 66,67. These findings are consistent with observations from Stopsack et al. and Brown and his colleagues. In breast cancer and PC, SQLE overexpression is more common and is an independent prognostic factor for poor prognosis 68,69. Given that SQLE may be oncogenic in a growing number of cancers, molecules targeting the SQLE signaling axis may be effective therapeutics for the treatment of malignancies.

### OSC

Oxidative squalene cyclase (OSC) catalyzes the transformation of the linear triterpene (3S)-2,3-oxidos-qualene into cyclic compounds 70. A previous study investigated the role of this post-squalene enzyme, which is involved in the cholesterol biosynthesis pathway, in regulating tumor angiogenesis and metastatic dissemination in mouse models of cancer. The authors showed that Ro 48-8071, a selective inhibitor of OSC 71, inhibited tumor growth in a spontaneous mouse model of pancreatic cancer and two metastatic mouse models. Remarkably, OSC inhibition suppressed metastasis formation in both the HCT116 and HPAF-II models. OSC inhibition specifically interfered with the PI3K pathway 72. Ro 48-8071 also potently reduced breast cancer cell viability, especially in human breast cancer cells. The administration of Ro 48-8071 to mice with tumor xenografts prevented tumor growth with no apparent toxicity. Ro 48-8071 had no effect on the viability of normal human mammary cells 73. *In vivo*, Ro 48-8071 effectively inhibited the growth of human PC xenograft cells in an anti-castration setting, without any signs of toxicity in experimental animals. Importantly, Ro 48-8071 did not decrease the survival rate of normal prostate cells *in vitro* 74. Ro 48-8071 is a potent inhibitor of cancer cell proliferation.

### ABCA1

Adenosine triphosphate (ATP)-binding cassette (ABC) transporters are drug efflux pumps that can lead to multidrug resistance and to tumor treatment failure 75,76. ATP-binding cassette transporter A-1 (ABCA1) mediates the export of cholesterol and phospholipids to apolipoprotein A-I for HDL assembly 77. In addition, ABCA1 is involved in bidirectional sterol movement through the plasma membrane, and regulates cell cholesterol homeostasis 78.

Compared with the sensitive MDA-MB-231 breast cancer cells, the overexpression of *ABCA1*, a gene involved in the lipid removal pathway in drug-resistant M14 melanoma 79. In human PC biopsy specimens, the expression of *ABCA1* mRNA was approximately twice as high in the androgen-depleted treatment group than in benign prostatic hyperplasia or PC 80. Suppression of the cholesterol transporter ABCA1 inhibited ovarian cancer cell growth and migration *in vitro*. Additionally, the expression of ABCA transporters is correlated with poor outcomes in serous ovarian cancer 81. JNJ-26854165 is a new type of chemotherapy with p53 activation abilities 82,83. Interestingly, ABCA1 depletion increased sensitivity to JNJ-26854165 84.

ABCA1 exhibits anticancer activity that inhibits the expression of the *ABCA1* gene through functional mutations in oncogenes or cancer-specific ABCA1. In 2012, one excellent study by Smith et al. revealed that the anticancer activity of ABCA1 efflux is impaired after *ABCA1* gene expression is inhibited by either an oncogenic mutation or cancer-specific ABCA1 functional deletion mutation. ABCA1 deficiency in conjunction with the high cholesterol synthesis found in cancer cells can lead to increased mitochondrial cholesterol, thereby promoting cancer cell survival 19. In LNCaP cells, a DNA methylome analysis revealed that the promoter of *ABCA1* is markedly hypermethylated. These findings suggest that the loss of cancer-specific *ABCA1* hypermethylation and protein expression directly leads to elevated levels of cholesterol in cells, thereby contributing to tumor development 85. ABCA1 is also aberrantly expressed in colon cancer tissues and cells. Silencing ABCA1 or miR-183 promoted proliferation and inhibited apoptosis in colon cancer cells 86.

## Conclusions

In summary, cancer cells rely on cholesterol as a cellular building block for membrane formation and for the production of signaling molecules. This review highlights the importance of cholesterol, cholesterol transporters and metabolites, and key enzymes of cholesterol metabolism in cancer (Figure 1). The requirement of cholesterol for cancer cell proliferation reveals a potential cancer therapeutic target at multiple points within the cholesterol metabolism pathway to inhibit proliferation. Numerous chemical inhibitors for specific steps in the metabolism pathway already exist. Targeting cholesterol metabolism could be more a selective therapeutic modality for highly proliferative cells. Alternatively, cholesterol metabolism inhibitors could be utilized in a cell-specific and targeted manner. Cancer is a diverse set of diseases with various genetic changes. Cholesterol metabolism is also complex, with many different feedback mechanisms and regulatory points. In addition, most cholesterol metabolism enzymes have multiple isoforms, which may be connected to different lipid metabolism processes, cellular localization, or tissue distribution.

Successful treatments may depend on understanding specific metabolic abnormalities in certain types of cancer 87,88 (Figure 1). While some cholesterol metabolites contribute to cancer development and resistance, others have therapeutic potential. Further insight on cholesterol metabolism in cancer cells will allow us to take advantage of new and effective targets to improve the survival rate of cancer patients.

## Figures and Tables

**Figure 2:**
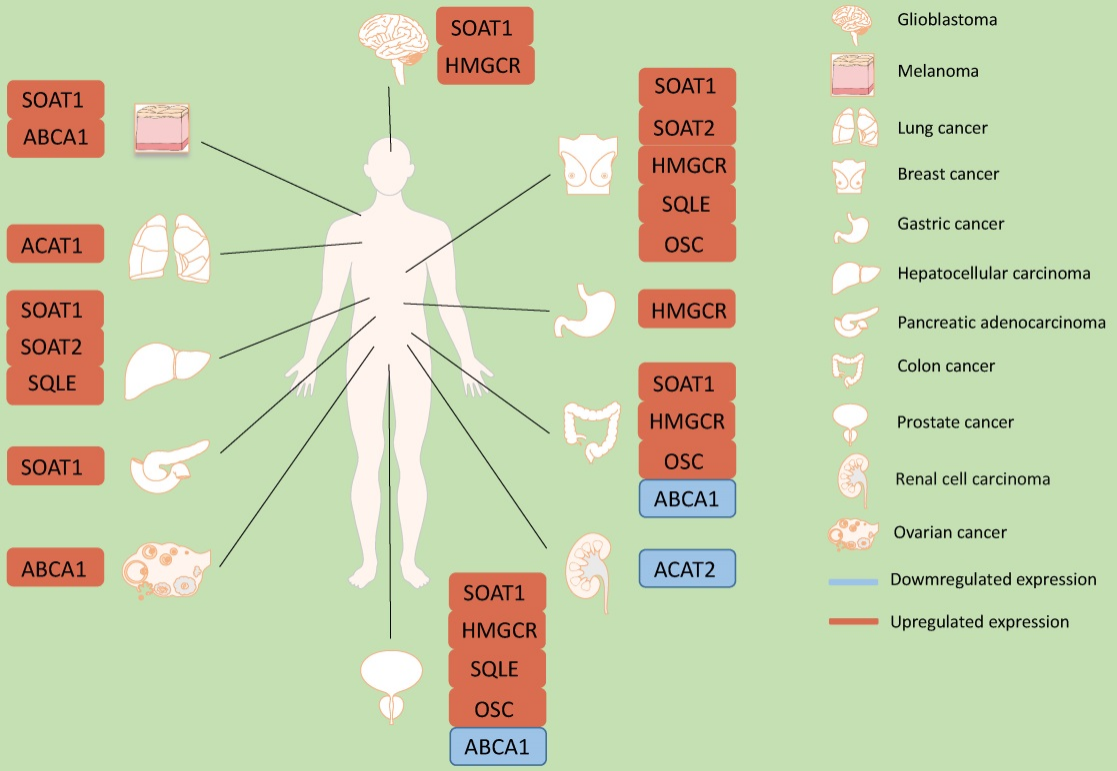
** Role of cholesterol synthesis enzymes in different kinds of cancers.** In glioblastoma, SOAT1 and HMGCR are up-regulated. In melanoma, ABCA1 and SOAT1 are up-regulated. In lung cancer, SOAT1 is up-regulated. And in breast cancer, SOAT1, SOAT2, HMGCR, SQLE and OSC are up-regulated. In gastric cancer, HMGCR is up-regulated. In hepatocellular carcinoma, SOAT2 and SQLE are up-regulated. In pancreatic adenocarcinoma, SOAT1 is up-regulated. In colon cancer, SOAT1, HMGCR and OSC are up-regulated. However, ABCA1 is down-regulated. In prostate cancer, SOAT1, HMGCR, SQLE and OSC are up-regulated. ABCA1 is down-regulated. In renal cell carcinoma, ACAT2 is up-regulated. In ovarian cancer, ABCA1 is up-regulated.

## Abbreviations

HDL: high-density lipoprotein
LDL: low-density lipoprotein
SREBPs: sterol regulatory element binding proteins
SR-BI: scavenger receptor class B, type I
PI3K: phosphatidylinositol 3-kinase
AKT: protein kinase B
CRC: colorectal cancer
ROS: reactive oxygen species
ER: estrogen receptor
PC: prostate cancer
ISC: intestinal stem cell
EC: endometrial cancer
NADPH: nicotinamide adenine dinucleotide phosphate
HBXIP: Hepatitis B X-interacting protein
FAS: fatty acid synthase
NR: nuclear receptor
SCAP: SREBP cleavage activation protein
ACAT: acetyl-coA acetyltransferase
AH: arecoline hydrobromide
PDC: pyruvate dehydrogenase complex
SOAT: sterol-o-acyltransferase (also named acyl-coA cholesterol acyltransferase)
CE: cholesteryl ester
SE: steryl ester
LD: lipoprotein-depleted
HCC: hepatocellular carcinoma
HMGCR: 3-hydroxy-3-methylglutaryl-CoA reductase
CSS: cancer-specific survival
HMGCS1: 3‐hydroxy‐3‐methylglutaryl‐CoA synthase 1
AML: acute myelocytic leukemia
BMP: bone morphogenetic protein
SQLE: squalene epoxidase
NAFLD: nonalcoholic fatty liver disease
OSC: oxidative squalene cyclase
ATP: adenosine triphosphate
ABCA1: ATP-binding cassette transporter A-1

## References

[1] Currie E, Schulze A, Zechner R, Walther TC, Farese RV Jr (2013). Cellular fatty acid metabolism and cancer. *Cell Metab*.

[2] Boroughs LK, DeBerardinis RJ (2015). Metabolic pathways promoting cancer cell survival and growth. *Nat Cell Biol*.

[3] Hanahan D, Weinberg RA (2011). Hallmarks of cancer: the next generation. *Cell*.

[4] DeBerardinis RJ, Thompson CB (2012). Cellular metabolism and disease: what do metabolic outliers teach us?. *Cell*.

[5] Huang C, Freter C (2015). Lipid metabolism, apoptosis and cancer therapy. *International journal of molecular sciences*.

[6] Gorin A, Gabitova L, Astsaturov I (2012). Regulation of cholesterol biosynthesis and cancer signaling. *Curr Opin Pharmacol*.

[7] Warburg O (1956). On the origin of cancer cells. *Science*.

[8] Luo X, Cheng C, Tan Z (2017). Emerging roles of lipid metabolism in cancer metastasis. *Molecular cancer*.

[9] Beloribi-Djefaflia S, Vasseur S, Guillaumond F (2016). Lipid metabolic reprogramming in cancer cells. *Oncogenesis*.

[10] Parrales A, Iwakuma T (2016). p53 as a Regulator of Lipid Metabolism in Cancer.

[11] Ribas V, Garcia-Ruiz C, Fernandez-Checa JC (2016). Mitochondria, cholesterol and cancer cell metabolism. *Clin Transl Med*.

[12] Merino Salvador M, Gomez de Cedron M, Moreno Rubio J (2017). Lipid metabolism and lung cancer. *Crit Rev Oncol Hematol*.

[13] Santos CR, Schulze A (2012). Lipid metabolism in cancer. *FEBS J*.

[14] Bathaie S Z AM, Azizian M (2017). Mevalonate Pathway and Human Cancers.

[15] Murai T (2015). Cholesterol lowering: role in cancer prevention and treatment. *Biological chemistry*.

[16] Jacobs RJ, Voorneveld PW, Kodach LL, Hardwick JC (2012). Cholesterol metabolism and colorectal cancers. *Curr Opin Pharmacol*.

[17] Kuzu OF, Noory MA, Robertson GP (2016). The Role of Cholesterol in Cancer. *Cancer Res*.

[18] Poulose N, Amoroso F, Steele RE, Singh R, Ong CW, Mills IG (2018). Genetics of lipid metabolism in prostate cancer. *Nat Genet*.

[19] Smith B, Land H (2012). Anticancer activity of the cholesterol exporter ABCA1 gene. *Cell reports*.

[20] Pietrocola F, Galluzzi L, Bravo-San Pedro JM, Madeo F, Kroemer G (2015). Acetyl coenzyme A: a central metabolite and second messenger. *Cell Metab*.

[21] Silvente-Poirot S PM (2014). Cancer. Cholesterol and cancer, in the balance.

[22] Guo Y, Fan Y, Zhang J (2015). Perhexiline activates KLF14 and reduces atherosclerosis by modulating ApoA-I production. *J Clin Invest*.

[23] Hall AM, Finck BN (2017). ChREBP refines the hepatic response to fructose to protect the liver from injury. *J Clin Invest*.

[24] Hoogerland JA, Lei Y, Wolters JC (2019). Glucose-6-Phosphate Regulates Hepatic Bile Acid Synthesis in Mice.

[25] Wang B, Tontonoz P (2018). Liver X receptors in lipid signalling and membrane homeostasis. *Nat Rev Endocrinol*.

[26] Meinsohn MC, Smith OE, Bertolin K, Murphy BD (2019). The Orphan Nuclear Receptors Steroidogenic Factor-1 and Liver Receptor Homolog-1: Structure, Regulation, and Essential Roles in Mammalian Reproduction. *Physiol Rev*.

[27] Horton JD, Goldstein JL, Brown MS (2002). SREBPs: activators of the complete program of cholesterol and fatty acid synthesis in the liver. *The Journal of clinical investigation*.

[28] Zhao Y, Li H, Zhang Y (2016). Oncoprotein HBXIP Modulates Abnormal Lipid Metabolism and Growth of Breast Cancer Cells by Activating the LXRs/SREBP-1c/FAS Signaling Cascade. *Cancer Res*.

[29] Gabitova L, Gorin A, Astsaturov I (2014). Molecular pathways: sterols and receptor signaling in cancer. *Clin Cancer Res*.

[30] Kadoya M, Hida A, Hashimoto Maeda M (2016). Cancer association as a risk factor for anti-HMGCR antibody-positive myopathy. *Neurol Neuroimmunol Neuroinflamm*.

[31] Allenbach Y, Keraen J, Bouvier AM (2016). High risk of cancer in autoimmune necrotizing myopathies: usefulness of myositis specific antibody. *Brain: a journal of neurology*.

[32] Bengtsson E NP, Wangefjord S (2014). HMG-CoA reductase expression in primary colorectal cancer correlates with favourable clinicopathological characteristics and an improved clinical outcome.

[33] Chushi L, Wei W, Kangkang X, Yongzeng F, Ning X, Xiaolei C (2016). HMGCR is up-regulated in gastric cancer and promotes the growth and migration of the cancer cells. *Gene*.

[34] Qiu Z, Yuan W, Chen T (2016). HMGCR positively regulated the growth and migration of glioblastoma cells. *Gene*.

[35] Ashida S KC, Inoue K (2017). Stromal regulation of prostate cancer cell growth by mevalonate pathway enzymes HMGCS1 and HMGCR.

[36] Singh R YV, Kumar S (2015). MicroRNA-195 inhibits proliferation, invasion and metastasis in breast cancer cells by targeting FASN, HMGCR, ACACA and CYP27B1.

[37] Clendening JW, Penn LZ (2012). Targeting tumor cell metabolism with statins. *Oncogene*.

[38] Gazzerro P, Proto MC, Gangemi G (2012). Pharmacological actions of statins: a critical appraisal in the management of cancer. *Pharmacol Rev*.

[39] Nielsen SF, Nordestgaard BG, Bojesen SE (2012). Statin use and reduced cancer-related mortality. *N Engl J Med*.

[40] Li AJ, Elmore RG, Chen IY, Karlan BY (2010). Serum low-density lipoprotein levels correlate with survival in advanced stage epithelial ovarian cancers. *Gynecol Oncol*.

[41] Cai H, Zhang G, Wang Z, Luo Z, Zhou X (2015). Relationship between the use of statins and patient survival in colorectal cancer: a systematic review and meta-analysis. *PLoS One*.

[42] Mamtani R, Lewis JD, Scott FI (2016). Disentangling the Association between Statins, Cholesterol, and Colorectal Cancer: A Nested Case-Control Study. *PLoS Med*.

[43] Hwang KE, Na KS, Park DS (2011). Apoptotic induction by simvastatin in human lung cancer A549 cells via Akt signaling dependent down-regulation of survivin. *Invest New Drugs*.

[44] Voorneveld PW, Reimers MS, Bastiaannet E (2017). Statin Use After Diagnosis of Colon Cancer and Patient Survival. *Gastroenterology*.

[45] Wang Y, Xu SL, Wu YZ (2013). Simvastatin induces caspase-dependent apoptosis and activates P53 in OCM-1 cells. *Exp Eye Res*.

[46] Parrales A, Ranjan A, Iyer SV (2016). DNAJA1 controls the fate of misfolded mutant p53 through the mevalonate pathway. *Nat Cell Biol*.

[47] Balasse EO, Fery F (1989). Ketone body production and disposal: effects of fasting, diabetes, and exercise. *Diabetes/metabolism reviews*.

[48] Haapalainen AM, Merilainen G, Pirila PL, Kondo N, Fukao T, Wierenga RK (2007). Crystallographic and kinetic studies of human mitochondrial acetoacetyl-CoA thiolase: the importance of potassium and chloride ions for its structure and function. *Biochemistry*.

[49] Haapalainen AM, Merilainen G, Wierenga RK (2006). The thiolase superfamily: condensing enzymes with diverse reaction specificities. *Trends in biochemical sciences*.

[50] Fan J, Shan C, Kang HB (2014). Tyr phosphorylation of PDP1 toggles recruitment between ACAT1 and SIRT3 to regulate the pyruvate dehydrogenase complex. *Molecular cell*.

[51] Fan J, Lin R, Xia S (2016). Tetrameric Acetyl-CoA Acetyltransferase 1 Is Important for Tumor Growth. *Molecular cell*.

[52] Zhao Z, Lu J, Han L, Wang X, Man Q, Liu S (2016). Prognostic significance of two lipid metabolism enzymes, HADHA and ACAT2, in clear cell renal cell carcinoma. *Tumour Biol*.

[53] Pramfalk C, Davis MA, Eriksson M, Rudel LL, Parini P (2005). Control of ACAT2 liver expression by HNF1. *Journal of lipid research*.

[54] Rogers MA, Liu J, Song BL, Li BL, Chang CC, Chang TY (2015). Acyl-CoA:cholesterol acyltransferases (ACATs/SOATs): Enzymes with multiple sterols as substrates and as activators. *J Steroid Biochem Mol Biol*.

[55] Jiang Y, Sun A, Zhao Y (2019). Proteomics identifies new therapeutic targets of early-stage hepatocellular carcinoma. *Nature*.

[56] Saraon P CD, Musrap N (2013). Quantitative proteomics reveals that enzymes of the ketogenic pathway are associated with prostate cancer progression.

[57] Li J, Gu D, Lee SS (2016). Abrogating cholesterol esterification suppresses growth and metastasis of pancreatic cancer. *Oncogene*.

[58] de Medina P, Genovese S, Paillasse MR (2010). Auraptene is an inhibitor of cholesterol esterification and a modulator of estrogen receptors. *Mol Pharmacol*.

[59] Lee S S LJ, Tai J N (2015). Avasimibe Encapsulated in Human Serum Albumin Blocks Cholesterol Esterification for Selective Cancer Treatment.

[60] Paillasse MR, de Medina P, Amouroux G, Mhamdi L, Poirot M, Silvente-Poirot S (2009). Signaling through cholesterol esterification: a new pathway for the cholecystokinin 2 receptor involved in cell growth and invasion. *Journal of lipid research*.

[61] Antalis CJ, Arnold T, Rasool T, Lee B, Buhman KK, Siddiqui RA (2010). High ACAT1 expression in estrogen receptor negative basal-like breast cancer cells is associated with LDL-induced proliferation. *Breast Cancer Res Treat*.

[62] Yang W, Bai Y, Xiong Y (2016). Potentiating the antitumour response of CD8(+) T cells by modulating cholesterol metabolism. *Nature*.

[63] Lu M, Hu XH, Li Q (2013). A specific cholesterol metabolic pathway is established in a subset of HCCs for tumor growth. *J Mol Cell Biol*.

[64] Huang Y, Jin Q, Su M (2017). Leptin promotes the migration and invasion of breast cancer cells by upregulating ACAT2. *Cell Oncol (Dordr)*.

[65] Gill S, Stevenson J, Kristiana I, Brown AJ (2011). Cholesterol-dependent degradation of squalene monooxygenase, a control point in cholesterol synthesis beyond HMG-CoA reductase. *Cell Metab*.

[66] Dabin Liu CCW, Li Fu, Huarong Chen, Liuyang Zhao, Chuangen Li, Yunfei Zhou, Yanquan Zhang, Weiqi Xu, Yidong Yang, Bin Wu, Gong Cheng, Paul Bo-San Lai, Nathalie Wong JJYS, Jun Yu (2018). Squalene epoxidase drives NAFLD-induced hepatocellular carcinoma and is a pharmaceutical target.

[67] Chua NK, Coates HW, Brown AJ (2018). Cholesterol, cancer, and rebooting a treatment for athlete's foot.

[68] Brown DN, Caffa I, Cirmena G (2016). Squalene epoxidase is a bona fide oncogene by amplification with clinical relevance in breast cancer. *Sci Rep*.

[69] Stopsack KH, Gerke TA, Sinnott JA (2016). Cholesterol Metabolism and Prostate Cancer Lethality. *Cancer Res*.

[70] Lenhart A, Weihofen WA, Pleschke AE, Schulz GE (2002). Crystal structure of a squalene cyclase in complex with the potential anticholesteremic drug Ro48-8071. *Chemistry & biology*.

[71] Morand OH, Aebi JD, Dehmlow H (1997). Ro 48-8.071, a new 2,3-oxidosqualene:lanosterol cyclase inhibitor lowering plasma cholesterol in hamsters, squirrel monkeys, and minipigs: comparison to simvastatin. *Journal of lipid research*.

[72] Maione F, Oliaro-Bosso S, Meda C (2015). The cholesterol biosynthesis enzyme oxidosqualene cyclase is a new target to impair tumour angiogenesis and metastasis dissemination. *Sci Rep*.

[73] Liang Y B-WC, Aebi J D (2014). Cholesterol biosynthesis inhibitors as potent novel anti-cancer agents: suppression of hormone-dependent breast cancer by the oxidosqualene cyclase inhibitor RO 48-8071.

[74] Liang Y, Mafuvadze B, Aebi JD, Hyder SM (2016). Cholesterol biosynthesis inhibitor RO 48-8071 suppresses growth of hormone-dependent and castration-resistant prostate cancer cells. *Onco Targets Ther*.

[75] Deeley RG, Westlake C, Cole SP (2006). Transmembrane transport of endo- and xenobiotics by mammalian ATP-binding cassette multidrug resistance proteins. *Physiol Rev*.

[76] Borel F, Han R, Visser A (2012). Adenosine triphosphate-binding cassette transporter genes up-regulation in untreated hepatocellular carcinoma is mediated by cellular microRNAs. *Hepatology*.

[77] Oram JF, Heinecke JW (2005). ATP-binding cassette transporter A1: a cell cholesterol exporter that protects against cardiovascular disease. *Physiol Rev*.

[78] Yamauchi Y, Iwamoto N, Rogers MA (2015). Deficiency in the Lipid Exporter ABCA1 Impairs Retrograde Sterol Movement and Disrupts Sterol Sensing at the Endoplasmic Reticulum. *J Biol Chem*.

[79] Bachmeier BE, Iancu CM, Killian PH (2009). Overexpression of the ATP binding cassette gene ABCA1 determines resistance to Curcumin in M14 melanoma cells. *Molecular cancer*.

[80] Sekine Y, Demosky SJ, Stonik JA (2010). High-density lipoprotein induces proliferation and migration of human prostate androgen-independent cancer cells by an ABCA1-dependent mechanism. *Mol Cancer Res*.

[81] Hedditch EL, Gao B, Russell AJ (2014). ABCA transporter gene expression and poor outcome in epithelial ovarian cancer.

[82] Smith MA, Gorlick R, Kolb EA (2012). Initial testing of JNJ-26854165 (Serdemetan) by the pediatric preclinical testing program. *Pediatric blood & cancer*.

[83] Kojima K, Burks JK, Arts J, Andreeff M (2010). The novel tryptamine derivative JNJ-26854165 induces wild-type p53- and E2F1-mediated apoptosis in acute myeloid and lymphoid leukemias. *Mol Cancer Ther*.

[84] Jones RJ, Gu D, Bjorklund CC (2013). The novel anticancer agent JNJ-26854165 induces cell death through inhibition of cholesterol transport and degradation of ABCA1. *J Pharmacol Exp Ther*.

[85] Lee BH, Taylor MG, Robinet P (2013). Dysregulation of cholesterol homeostasis in human prostate cancer through loss of ABCA1. *Cancer Res*.

[86] Bi DP, Yin CH, Zhang XY, Yang NN, Xu JY (2016). MiR-183 functions as an oncogene by targeting ABCA1 in colon cancer. *Oncol Rep*.

[87] Thurnher M, Gruenbacher G, Nussbaumer O (2013). Regulation of mevalonate metabolism in cancer and immune cells. *Biochim Biophys Acta*.

[88] Hay N (2016). Reprogramming glucose metabolism in cancer: can it be exploited for cancer therapy?. *Nat Rev Cancer*.

